# Editorial: The role of conversational AI in higher education

**DOI:** 10.3389/frai.2025.1602037

**Published:** 2025-04-24

**Authors:** Pauldy C. J. Otermans, Stavros Demetriadis, Deborah Richards

**Affiliations:** ^1^Department of Psychology, Brunel University of London, Uxbridge, United Kingdom; ^2^Department of Informatics, Aristotle University of Thessaloniki, Thessaloniki, Greece; ^3^School of Computing, Macquarie University, Sydney, NSW, Australia

**Keywords:** artificial intelligence, AI, higher education, conversational AI, teaching, learning

As conversational Artificial Intelligence (AI) technologies rapidly evolve, their influence on Higher Education (HE) becomes increasingly profound. This editorial explores how recent research on conversational AI contributes to the growing discourse surrounding its role in academic settings, specifically in teaching, learning, and student engagement. The six articles within this Research Topic provide a range of insights into the effectiveness, challenges, and future potential of AI in HE. These studies collectively highlight the promise of AI, while underscoring the need for careful consideration of its limitations and the impact on educational practices.

## AI-Powered pedagogical feedback

One Arguedas et al. examines the impact of AI in the form of Affective Pedagogical Tutors (APT) that delivers both cognitive and affective feedback to students in a web design course. In a study with 115 students, the research compared the effectiveness of feedback provided by an AI tutor vs. a human instructor. The findings revealed that while cognitive feedback from the AI significantly enhanced student learning outcomes, the affective feedback was less impactful, suggesting that further refinement is necessary in the design of emotional support mechanisms within AI systems. This study highlights the potential of AI to contribute to improved student performance, particularly when it is able to offer personalized and immediate feedback. However, it also underscores the need for ongoing research to optimize AI's role in affective support, a dimension that requires more nuanced design to be effective.

## AI teachers across borders

Another Aditya et al. explores the potential of AI teachers in enhancing employability skills across three countries. Using a fine-tuned Large Language Model (LLM) called OIMISA, this research provides a 9-lesson course on employability and transferable skills, enrolling 207 students from various institutions. The results indicated a high engagement rate, with over 47% completion and strong student satisfaction. These findings suggest that AI teachers may offer a viable alternative to traditional online learning platforms like MOOCs, providing a more personalized and interactive learning experience. This research further strengthens the argument that AI can bridge educational gaps across diverse international contexts, ensuring that learning is more accessible and tailored to the needs of individual students.

## AI tools and student perception

While much of the discourse has focused on the technical capabilities of AI, another Thomson et al. shifts the focus to students' perceptions of AI tools in academia. By surveying 453 students in the UK and conducting focus groups, the research examined students' familiarity with AI tools, their views on AI's potential use in education, and their knowledge of university AI policies. The results showed a strong desire among students to learn more about AI and to receive dedicated support in integrating these tools into their coursework. However, there was also a lack of awareness about existing AI policies at their institutions, pointing to a gap in communication between universities and students. The study emphasizes that as AI technologies become integral to academic practice, universities must adopt a more comprehensive approach to communicating their AI policies, ensuring that students are adequately informed about how to use these tools responsibly and effectively.

## Evaluating AI's performance in assessments

The increasing adoption of AI for academic assessments is also explored in a Al Mashagbeh et al. that evaluates the performance of ChatGPT and Google Bard in answering various question types in engineering and health sciences. The study found that ChatGPT-4 outperformed both ChatGPT-3.5 and Google Bard in problem-solving and accuracy, particularly excelling at true/false questions. However, the AI models struggled with simple calculations and certain types of multiple-choice questions, especially in health sciences. The findings highlight that while AI tools such as ChatGPT demonstrate considerable promise for supporting educational assessments, they still have limitations that require human oversight. This research reinforces the notion that AI can be a powerful tool in education, but its use must be carefully calibrated to avoid misapplication, particularly when it comes to tasks that require high levels of accuracy, such as calculations.

## The changing landscape of computing education

As conversational AI becomes more prevalent in educational settings, its implications for computing education are also a topic of critical debate. Sengul et al. explore the use of AI in software engineering education reveals a significant gap between the rapid adoption of AI in industry and its slower integration into HE. The research calls for a more strategic approach to incorporating AI tools into computing curricula, pointing out that while software engineering practices have embraced AI, academic institutions have been slower to adapt. This disconnect suggests that the current approach to computing education may need to evolve to better align with the demands of the industry, where AI is increasingly becoming a foundational tool for software development. The study suggests that AI could play a crucial role in shaping the future of computing education by helping students gain the skills needed to thrive in a tech-driven job market.

## AI and moral decision-making

The final in this Research Topic by Proksch et al. takes a different approach by examining students' perceptions of AI in the context of moral decision-making. A controlled experiment involving 164 participants tested the perception of AI-generated texts on moral and technological topics. The study found that students consistently devalued AI compared to human authors, particularly when it came to texts on moral issues. These results align with algorithm aversion theory, suggesting that while students may embrace AI for technological tasks, they are less comfortable trusting AI for moral or ethical decisions. This highlights a critical challenge for the wider acceptance of AI in education: how to address the concerns surrounding the role of AI in making moral judgments, especially when it comes to sensitive academic tasks that involve ethical considerations.

## Conclusion

The contributions in this Research Topic collectively reflect the potential and challenges associated with integrating conversational AI into HE. From enhancing learning outcomes through personalized feedback to offering scalable teaching solutions across borders, AI offers numerous benefits. However, the findings also underscore the need for careful consideration of its limitations, including the accuracy of AI-generated content, the lack of familiarity with AI policies among students, and the complex ethical issues surrounding its use.

The studies presented in this Research Topic demonstrate that while AI has the potential to transform HE, its implementation must be accompanied by thoughtful policy development, continuous refinement of AI tools, and a balanced approach that addresses both the opportunities and challenges that come with the integration of AI in academia. As AI continues to evolve, it is crucial that educators, students, and institutions collaborate to harness its full potential while ensuring that its use is ethical, transparent, and aligned with the broader goals of education.

This editorial has framed the aims and outcomes of the six articles in the broader context of the role conversational AI is playing in reshaping HE. As AI tools become more ubiquitous, the ongoing discourse and research on their application in educational settings will remain vital in navigating the complexities of these technologies.

